# Effects of Corneal Stromal Lens Collagen Cross-Linking Regraft on Corneal Biomechanics

**DOI:** 10.1155/2022/8372156

**Published:** 2022-06-08

**Authors:** Rong Shi, Weize Wang, Yu Che, Shaorong Linghu, Taixiang Liu

**Affiliations:** Department of Myopia and Cataract Center, The Affiliated Hospital of Zunyi Medical University, Zunyi, Guizhou, China

## Abstract

**Background:**

Corneal collagen cross-linking (CXL) therapy, a method that uses a combination of riboflavin and ultraviolet-A light (UVA), can promote the formation of covalent cross-linking of amino acid residues of corneal collagen and enhance the hardness of the cornea. In this study, we explored the effects of corneal stromal lens collagen cross-linking regraft on corneal biomechanics.

**Methods:**

A total of 15 New Zealand white rabbits were divided into 3 groups: normal control group (group A), SMILE + uncross-linked lens implantation group (Group B), and SMILE + cross-linking lens implantation group (group C). The design parameters of SMILE surgery were as follows: the corneal cap was 120 um thick, the lens diameter was 6.5 mm, and the diopter was -6.0D. Riboflavin and ultraviolet-A (UVA) were used as corneal stromal lens CXL, which was implanted into the allogeneic rabbit corneal stromal bag 24 hours after the operation. Postoperative corneal thickness (CCT), refraction, AS-OCT, and corneal biomechanics were performed before and then at 1 and 3 months after the operation.

**Results:**

All corneas appeared transparent and smooth 3 months after surgery. The corneal thicknesses of both group B and group C were lower than those before the operation. The corrected refraction of group B and group C after lens implantation was also lower than the expected corrected power; there was no significant difference between the two groups (*P* > 0.05). AS-OCT results showed an uneven surface and thickness of the corneal stromal lens in two eyes of group B. Moreover, corneal elastic deformation increased with intraocular pressure in each group; displacement from large to small was group B > group C and > group A. The creep from large to small was group B > group C > group A. The fiberboard layers of groups B and C were disordered, and there were a few autophagosomes in the fibroblasts of group B by transmission electron microscopy (TEM).

**Conclusions:**

Allograft graft of corneal stromal lens collagen cross-linked can significantly increase the biomechanical properties of the cornea.

## 1. Introduction

The cornea is an important element of the ocular refractive system that protects the structures inside the eye and contributes to the eye's refractive power. Corneal disease is a serious condition that causes distortion, scarring, and clouding and, in some severe cases, may lead to blindness. Corneal transplantation is the most widely used approach to treat corneal disease [1]. Nevertheless, the lack of donor materials limits its use, especially when treating keratopathy [2]. Thus, searching for new methods is urgently required.

The recent development of small incision lenticule extraction (SMILE) surgery and SMILE-derived corneal stromal lens has shown promising effects in treating myopia (nearsightedness) and irregularly shaped cornea [3, 4]. SMILE uses a femtosecond laser to regulate the thickness and size of the lens [5, 6]. Its safety and effectiveness have been demonstrated in several studies. Currently, SMILE is considered the main treatment for myopia [6], while its effect and safety in the treatment of hyperopia [7, 8], corneal ulcer perforation [9], keratoconus [10, 11], and other diseases [12, 13] have been extensively investigated. Yet, due to differences in the thinness, low hardness, weak strength, and damage of the single-lens, not all corneal stroma lenses have the same quality. A recent study suggested using superposition transplantation of multilayer lenses to improve the characteristics of weak and easily deformed lenses in the process of implantation. However, because the lens can easily move, angle, and crimp during the suturing process of a multilayer lens, surgery may be challenging and may lead to postoperative astigmatism and graft opacity. Moreover, some studies suggested the use of post-articular biological adhesive multi-disc lens for the treatment of corneal perforation; yet, after implantation, they noticed a clear boundary between the two lenses, which affected the healing and refractive state of the lens [2]. Farsightedness, presbyopia, and early keratoconus are the major limitations when using multilayer lens implantation to correct refractive corneal disease. Moreover, the thickness of the combined lens is too thick, which is not resistant to the implantation of the eye bag. Therefore, it is essential to enhance the hardness of the monolithic lens and improve its biomechanical properties.

Corneal collagen cross-linking (CXL) therapy has been attracting increasing attention in ophthalmological research over the last ten years. This method, which uses a combination of riboflavin and ultraviolet-A light (UVA), produces free radicals to promote the formation of covalent cross-linking of amino acid residues of corneal collagen and enhances the hardness of the cornea [14–16]. Treating iatrogenic corneal dilatation or early keratoconus with this approach has achieved a good clinical effect [17]. Yet, so far, no studies have reported on the effect of cross-linking of corneal stromal lens collagen.

In this study, we explored the effects of corneal stromal lens collagen cross-linking regraft on corneal biomechanics. Our results showed that the biomechanical properties and hardness of the CXL lens were significantly increased, and the anti-edema ability was also enhanced. This study provides a basis for the full application of SMILE lenses in clinic.

## 2. Materials and Methods

### 2.1. Animals and Grouping

A total of 15 healthy New Zealand white rabbits weighing 2.5–3.5 kg were obtained from XX. All the animals were housed in an environment with a temperature of 22 ± 1°C, relative humidity of 50 ± 1%, and a light/dark cycle of 12/12 hr. All animal studies (including the mice euthanasia procedure) were done in compliance with the regulations and guidelines of Affiliated Hospital of Zunyi Medical University institutional animal care and conducted according to the AAALAC guidelines (approval number [KLLY(A)-2019-074]).

Rabbits were randomly divided into 3 groups (*n* = 5/group): normal control group (group A), SMILE + uncross-linked lens implantation group (group B), and SMILE + cross-linking lens implantation group (group C). The right eye was selected as the experimental eye in each group.

### 2.2. SMILE Surgery

The concentration of pentobarbital sodium (30 g·L-1 and 1 mL) was intravenously injected into the ear of the rabbits 30 minutes before surgery to induce anesthesia. Normal saline was used for flushing the right conjunctival sac, and propimecaine (5 g/L) was used as eye drops.

The design parameters of SMILE surgery were as follows: corneal cap thickness of 120 um, diameter of 7.5 mm, corneal incision of 4 mm, lens diameter of 6.5 mm, adding matrix of 10 um, and diopter of −6.0 D. The following are details about the major procedures: (a) the cone was attached, and the eyeball was centered and fixed with negative pressure; (b) femtosecond laser scanning was performed according to the predetermined procedure; (c) the stromal lens was completely disconnected and removed. Finally, the lens was placed in a refrigerated tube filled with glycerin and marked.

### 2.3. Corneal Stromal Lens CXL

The lens was immersed in 0.1% riboflavin solution for 25 min. The illumination intensity was 3 mW/cm2. The irradiation time was 30 min by UVA ray irradiation (wavelength 370 nm, Zhuhai Tianhui Electronics Co Ltd, Zhuhai, China).

### 2.4. Lens Transplantation

**Figure 1:**
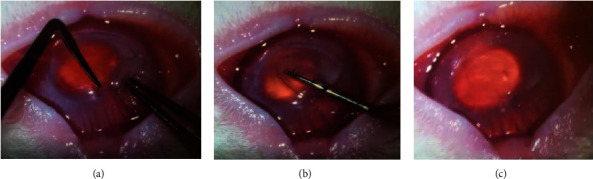
Schematic diagram of lens transplantation.

Rabbits were anesthetized using the same method as explained above. A microseparator was then used to separate the corneal cap, and a stromal layer of SMILE operation and a corneal stromal bag were formed. The lens was implanted into the corneal stromal bag (Figure 1).

### 2.5. Clinical Observation Index

The inflammatory reaction of the ocular surface was examined by slit-lamp microscopy. Corneal curvature was examined using pentacam analysis. Postoperative corneal thickness (CCT) was measured by ultrasound. Healing of implant and implant bed was examined by AS-OCT, and refraction was examined by retinoscopy.

### 2.6. Whole Eye Dilation Test

A self-made experimental device was used to fix the optic nerve end down on the platform with biological glue. A 25 G needle was inserted into the vitreous cavity through a scleral puncture from the optic nerve end to the equatorial part. The end of the 25 G needle was connected to the adjustable height hose to adjust the intraocular pressure through the lifting hose. The pressure was recorded with a Dino-Lite digital microscopic system (3411, Anmo Co, Taiwan). The displacement of corneal vertex was recorded when the intraocular pressure was 15 mmHg, 20 mmHg, 25 mmHg, 30 mmHg, 35 mmHg, 40 mmHg, 45 mmHg, and 50 mmHg. The intraocular pressure (100 mmHg) was maintained for 30 min to observe the corneal vertex displacement.

### 2.7. Electron Microscopy Examination

The ultrastructure of the cornea was observed by electron microscopy.

### 2.8. Statistical Analysis

SPSS17.0 software (SPSS Inc, Chicago-ILL, IL, USA) was used for data analysis in this study. The experimental data were expressed as mean ± standard deviation (SD). The data were analyzed by repeated-measures analysis of variance. *P* < 0.05 was considered statistically significant.

## 3. Results

### 3.1. Slit-Lamp Microscope Examination of Corneas in Different Groups

One month after surgery, all corneas appeared transparent and smooth under the slit-lamp microscopy. In group B, the boundary of the corneal stromal lens was obvious, and the edge was irregular. In group C, the boundary was faintly visible, and the edge was regular.

**Figure 2:**
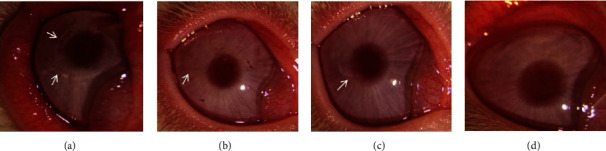
Postoperative representative slit lamp microscope images of group B and group C. (a) One month after the operation, the boundary of the corneal stromal lens in group B was obvious and the edge was irregular (white arrow). (b) The boundary in group C lens was faintly visible, and the edge was regular (white arrow). (c, d) Three months after the operation, the corneal stromal lenses in group B and C were basically fixed to the adjacent corneal stroma without obvious boundary.

Three months after the operation, the corneal stromal lenses in group B and C were basically fixed to the adjacent corneal stroma without obvious boundary (Figure 2).

### 3.2. Comparison of CCT in Different Groups

The CCT in group B and group C was lower 3 months after surgery than before the operation (all *P* < 0.05), and the difference between groups was not significant (*P* > 0.05) (Table 1).

### 3.3. Comparison of Refraction in Different Groups

The refraction of group B and C did not achieve the expected correction degree. The lens refraction was +6.0 D, while the actual correction degree after implantation was about +4.5 D. Also, there was no significant difference between the two groups (*P* > 0.05) (Table 2).

### 3.4. AS-OCT

One month after the operation, uneven surface and thickness of the corneal stromal lens were seen in group B. On the contrary, the lens of group C showed a flat surface, uniform thickness, and enhanced lens imaging. In both groups, the corneal stromal lens was closely combined with the implant bed, and there was no gap.

**Figure 3:**
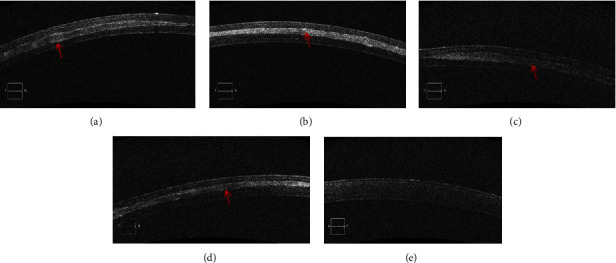
Postoperative OCT images in different groups. (a) One month after the operation, uneven surface and uneven thickness (red arrow) of the corneal stromal lens in group B. (b) Lens of group C showed flat surface, uniform thickness, and slightly enhanced lens development (red arrow). (c, d) Three months after the operation, the density of the lens decreased in group B and C (red arrow). (e) Control group.

Three months after the operation, the density of the lens decreased in both groups (Figure 3).

### 3.5. Biomechanical Analysis: Elastic Deformation and Corneal Creep Variables

**Figure 4:**
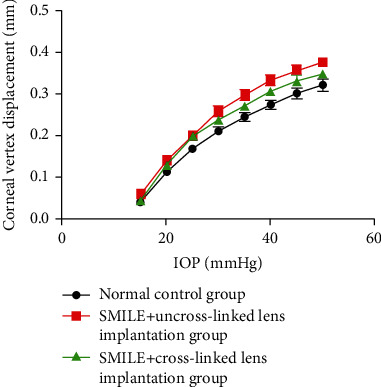
Corneal vertex displacement as a function of increased intraocular pressure.

Corneal vertex displacements at the intraocular pressure of 15 mmHg, 20 mmHg, 25 mmHg, 30 mmHg, 35 mmHg, 40 mmHg, 45 mmHg, and 50 mmHg were recorded. With the increase in intraocular pressure, the corneal vertex displacements in each group increased. The elasticity of the cornea increased with the increase of intraocular pressure and showed a nonlinear trend. The corneal displacement from large to small was group B > group C and > group A. There was no significant difference between group C and group A (*P* > 0.05), and the corneal displacement in group B was significantly higher than in group A (*P* < 0.05) (Figure 4).

**Figure 5:**
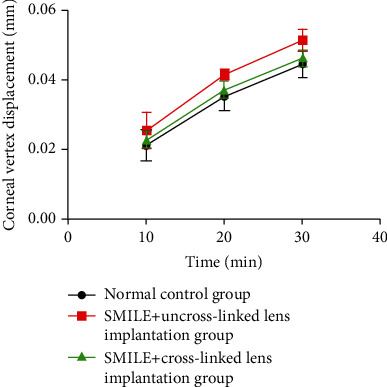
Changes of corneal vertex displacement at different time points. The intraocular pressure (100 mmHg) was maintained for 30 min to observe the corneal vertex displacement.

When the intraocular pressure was maintained at 100 mmHg for 30 min, the changes of corneal vertex displacement were observed as follows: group B > group C > group A. There was no significant difference between group C and group A (*P* > 0.05), and group B was significantly higher than group A (*P* < 0.05) (Figure 5).

### 3.6. Transmission Electron Microscopy (TEM)

**Figure 6:**
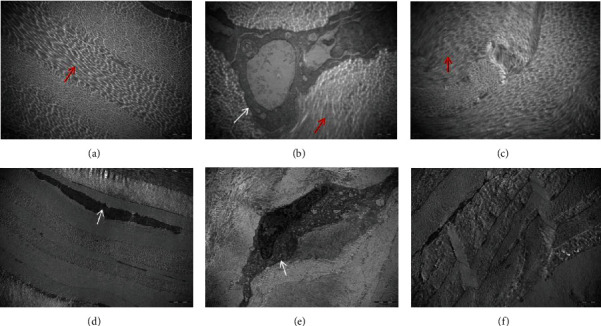
The postoperative TEM results of each group. (a, d) The lamellar structure of the cornea in group A was clearly visible (red arrow), and there were obviously flat and slender fibroblasts (white arrow) between the lamellar structures. (b, c) The fiberboard layers of group B (b) and C (c) were disordered (red arrow), and there were a few autophagosomes in the fibroblasts of group B. (c, f) The lamellar structure of group C was clear and more closely arranged (c, f). (a–c):10 K × 40, ruler: 500 nm, (d–f)10 K × 10 ruler: 2 um.

The lamellar structure of the cornea in group A was clearly visible, and there were obviously flat and slender fibroblasts between the lamellar structures. The fiberboard layers of groups B and C were disordered, and there were a few autophagosomes in the fibroblasts of group B. The lamellar structure of group C was clear and more closely arranged. The thickness of collagen fibers was consistent (Figure 6).

## 4. Discussion

Corneal stromal lenses from SMILE have been used in various ophthalmic applications, including refractive correction, biomechanical strengthening of the cornea, and stromal volume expansion. Clinically, they were found to be safe and effective for the treatment of corneal perforation, keratoconus [18], but also other corneal diseases [19]. However, the biological properties of the cornea after transplantation have been rarely reported. In this study, the corneal stromal lens derived from SMILE was implanted into the rabbit corneal stromal bag after CXL, and the healing situation, ocular refractive, corneal biomechanics, and other aspects were explored at 1 and 3 months after the operation.

We used 3 groups of rabbits in this study: the normal control group (group A), SMILE + uncross-linked lens implantation group (group B), and SMILE + cross-linking lens implantation group (group C). The edge of the lens in group B was clearly visible and irregular after implantation 1 month after surgery. Also, the boundary of the cross-linking lens was not obvious after lens implantation. We speculated that it might be because of the edema caused by the corneal stroma. However, the lens stiffness was increased, and the anti-edema ability was enhanced after cross-linking.

Three months after the operation, the corneal stromal lenses in group B and C were basically fixed to the adjacent corneal stroma without obvious boundary, while no infection, chronic interlamellar keratitis, opacity, or lens graft rejection was observed, suggesting that the cross-linking helps the lens to heal faster.

Our results showed that the thickness increase in group B and group C was lower than the expected thickness (112 um) after lens implantation, which is consistent with a previous study [20] and lower than that of the normal control group, which may be caused by the difference between the actual cutting thickness and the expected cutting thickness. Corneal thickness in the cross-linking group was slightly lower than that in group B, which was considered to be related to the tighter rearrangement of lens collagen and the enhanced anti-edema ability.

In this study, the lens refraction after implantation did not reach the expected correction degree. The lens refraction was +6.0D, while the actual correction degree after implantation was about +4.5D, which is consistent with previous studies [8, 21]. Zhen et al. [21] implanted +6.0 D bovine corneal lens into the corneal stromal bag after acellular treatment; 24 weeks after surgery, the refraction was only 1/3 of that before surgery. Moreover, Pradhan et al. [8] removed a + 10.0 d corneal stromal lens from the cornea of a patient with high near-myopia and implanted it into the stromal bag of a patient with + 12.0 d hyperopia. The postoperative refraction reached 1/2 of the expected correction. They believe this result is related to the remodeling of the corneal epithelium. In this study, the refraction of the cross-linking group (group C) was slightly lower than that of the uncross-linked group (group B); yet, the difference was not significant, indicating that the lens CXL had no significant effect on refraction.

A-OCT examination results showed that the surface and thickness of the stromal lens in group B were uneven 1 month after surgery. The surface of the lens in group C was flat, the thickness was uniform, and the lens imaging was enhanced. The two groups of the corneal stromal lens were closely combined with the implant bed. Three months after surgery, the implanted lenses in group B and group C were still clearly visible. The surface of the lens was more flat, and the imaging was significantly weaker than before in both groups. The lens was closely combined with the surrounding matrix, which indicated that CXL did not affect the healing between the lens and the host implant bed. Previous studies have shown that cross-linking effects of the FUR technique enable a stronger graft-recipient adhesion than conventional penetrating and anterior lamellar keratoplasty [21].

The cornea is a complex anisotropic composite with nonlinear elastic and viscoelastic properties [22]. The corneal stroma makes up 90% of corneal thickness and is the main contributor to the cornea's strength and transparency. This layer is composed of 250–400 stacked lamellae [23]. The lamellae are composed of type I/V collagen fibrils oriented in specific directions. The change in collagen structure usually indicates changes in collagen biomechanical properties [24]. The amount of collagen cross-linking fibers, the spatial arrangement, and the thickness of collagen fibers play a very important role in determining the biomechanical properties of the cornea [25]. In this study, we evaluated the biomechanical properties of the cornea by observing the elastic deformation and creep of the cornea. We found that, with the increase in intraocular pressure, the peak displacement of the cornea in each group increased. The elasticity of the cornea increased with the increase of intraocular pressure and showed a nonlinear trend. There was no significant difference between group C and group A, and group B was significantly greater than group A. The creep results showed that group B > group C > group A. There was no significant difference between group C and group A. Mattson et al. found that riboflavin/UVA treatment reduces expansion compared with that in both dextran-treated and untreated control corneas [25]. The biomechanics of the lens, which was further induced by CXL with riboflavin and UVA after implantation into the stromal bag of the cornea after SMILE, was close to that of the normal cornea, indicating that the resistance of the almost normal cornea can be achieved after the lens collagen is cross-linked, avoiding the complications caused by CXL in the whole cornea.

In this study, TEM showed autophagosomes in group B. The lamellar arrangement was more compact, the fibroblasts in the interlamellar space were reduced, and the thickness of collagen fibers was more uniform. In group C, the arrangement of collagen fibers in group B and group C was disordered. The healing of the grafts and the implant bed after cross-linking, the arrangement of fibers, and the ultrastructure indicated that the lens cross-linking implant could heal well, which provides strong evidence for the use of the lens. However, the arrangement of collagen fibers in the two groups was disordered. Since the postoperative visual quality may be affected by the structure of collagen fibers, further study is needed to determine whether the visual quality after lens implantation will be affected.

This study has some limitations. It has a small sample size and a short follow-up period. Thus, a larger sample study with longer observation period needs to be performed.

## 5. Conclusions

Our data suggest that the corneal stroma lens allogeneic transplantation can significantly increase the biomechanical properties of the cornea and enhance the ability of corneal resistance to tension and lens implantation after *in vitro* collagen cross-linking. The results have a certain clinical application value.

## Figures and Tables

**Table 1:** CCT data before and 3 months after lens implantation.

Group (*n* = 5)	Before operation (um)	After operation (um)	ΔCCT (um)	*P* value
Group B	365 ± 14.74	354 ± 13.63	11.40 ± 5.45	＜0.05
Group C	366 ± 10.38	351 ± 10.58	15.60 ± 9.63	＜0.05

**Table 2:** Changes in refraction in each group.

Group (*n* = 5)	Preoperative	Postoperative 1 month	Postoperative 3 months
Group A	3.35 ± 0.41	3.15 ± 0.52	3.03 ± 0.51
Group B	3.15 ± 0.29	4.20 ± 0.38	4.41 ± 0.29
Group C	3.25 ± 0.31	4.55 ± 0.37	4.75 ± 0.25
*F*	1.681	7.945	15.001
*P*	0.231	＜0.05	＜0.05

## Data Availability

The datasets generated during the current study are available from the corresponding author on reasonable request.
